# Identification of a cell-surface glycoprotein associated with normal mammary and extramammary epithelial cells.

**DOI:** 10.1038/bjc.1994.80

**Published:** 1994-03

**Authors:** S. A. Imam, M. R. Stampfer, A. Yilmaz, C. R. Taylor

**Affiliations:** Department of Pathology, University of Southern California School of Medicine, Los Angeles 90033.

## Abstract

**Images:**


*Br. J. Cancer* (1994), **69**, 439–444 © Macmillan Press Ltd., 1994

### Identification of a cell-surface glycoprotein associated with normal mammary and extramammary epithelial cells

S.A. Imam[1], M.R. Stampfer[2], A. Yilmaz[3] & C.R. Taylor[1]

[1]*Department of Pathology and Comprehensive Cancer Center, University of Southern California School of Medicine, Los Angeles, California 90033, USA;* [2]*Lawrence Berkeley Laboratory, University of California, Berkeley, California 94720, USA;* [3]*Swiss Institute for Experimental Cancer Research, CH-1066 Epalinges, Switzerland.*

**Summary** The goal of the study was to identify any normal genes that may become inactivated in malignant cells, with associated modifications or loss of gene products. Consequently, attempts were made to identify such products by generating monoclonal antibodies using an immune tolerisation–immunisation procedure. Using such a technique, a plasma membrane-associated glycoprotein with an apparent molecular weight of 92 kDa was identified. The glycoprotein was termed luminal epithelial antigen (LEA.92). The pattern of expression of LEA.92 was demonstrated by an indirect immunostaining technique. Using an *in vitro* model system representing various stages of breast oncogenesis, LEA.92 was detected on normal or immortalised mammary epithelial cell (MEC) lines which were dependent on epidermal growth factor (EGF) and anchorage formation for growth and non-tumorigenic in nude mice. In contrast, LEA.92 was undetectable on oncogenically transformed or established lines of mammary carcinoma cell lines which were independent of EGF or anchorage formation for growth and were highly tumorigenic. The results appear to suggest a correlation between the down-regulation of LEA.92 and the development of tumorigenicity in malignant MEC lines. Furthermore, the patterns of expression of LEA.92 on breast cells in tissue mirrored those of breast epithelial cells in cell cultures. LEA.92 was detected on the surface of normal but not malignant epithelial cells, which included breast, cervix, colon, lung, pancreas and stomach. LEA.92 appeared to be distinct from receptor for epidermal growth factor, antigens associated with milk fat globule membrane and the family of epithelium-specific keratins.

The hypopthesis that inactivation of tumour-suppressor genes plays an important role in the development of human malignancies has recently received considerable attention (Knudson, 1985; Stanbridge, 1985, 1987, 1990; Hansen & Cavenee, 1987; Klein, 1987; Friend *et al.*, 1988; Harris, 1988; Weinberg, 1989, 1991). To date, various such suppressor genes, namely retinoblastoma (RB1), Wilms' tumour (WT1), neurofibrosarcoma (NF1), APC, MCC, p53 and DCC genes, have been reported and their role in the suppression of tumour growth documented (Friend *et al.*, 1986, 1988; Lee *et al.*, 1987; Stanbridge, 1987, 1990; Yuen-Kai *et al.*, 1987; Lübbert *et al.*, 1988; Baker *et al.*, 1989; Eliyahu *et al.*, 1989; Takahasi *et al.*, 1989; Vogelstein *et al.*, 1989; Weinberg, 1989, 1991). In view of the success in defining such tumour-suppressor genes in the above organs, it seems logical to explore the possible presence of genes with similar functions in human breast cancer. Evidence for the loss of inactivation of oncosuppressor gene(s) in breast carcinomas comes from independent reports that describe allelic losses on several chromosomes, including 1q, 1p, 11p, 13q and 17p and 17q, in malignant cells from breast (Ali *et al.*, 1987; Lundberg *et al.*, 1987; Mackay *et al.*, 1988; Chen *et al.*, 1989; Genuardi *et al.*, 1989). Although relatively gross chromosomal defects have been observed (Pathak, 1992), the precise genes involved have not been determined and their specific gene products have not been identified. Therefore, the purpose of the study was to identify any normal breast cell products, encoded by genes that may become inactivated in the malignant counterpart, with associated loss of the products.

In order to achieve the above-stated goal, the procedure of tolerisation/immunisation (Imam *et al.*, 1990*a*) was modified to favour the production of antibody to any antigens present preferentially in normal mammary epithelial cells as compared with malignant breast cells. Immune tolerance to mammary epithelial cell lines (MCF.7 and MDA.MB.231 combined) was induced in neonatal mice (within 24 h of birth) prior to subsequent immunisation with an extract of normal breast tissue.

This paper reports the identification, generated by the immune tolerisation/immunisation protocol, of a plasma membrane-associated glycoprotein with an apparent molecular weight of 92 kDa (LEA.92) coexpressed on normal but not malignant epithelial cells in mammary and extra-mammary tissues and cell lines of normal mammary epithelium.

### Materials and methods

### *Cell lines*

The established human cell lines employed in this study were obtained from the American Type Culture Collection, Rockville, MD, USA (Table I). These cell lines were cultured in DMEM supplemented with 100 units $ml^{-1}$ penicillin, 10 $\mu g\ ml^{-1}$ insulin and 10% (v/v) fetal calf serum. In addition to using the above cell lines, a model system that consists of a normal human mammary epithelial cell (HMEC) line, designated 184 (Hammond *et al.*, 1984; Stampfer, 1985), immortalised MEC lines (designated 184A1 and 184B5) established from 184 cells by exposure to benzo-[*a*]pyrene (Stampfer & Bartley, 1985; Walen & Stampfer, 1989) and a transformed cell line (designated 184A1N1-T-D10) obtained from 184A1 by exposure to oncogenes (Clark *et al.*, 1988), reflecting various steps of neoplastic tranformation, was analysed for the expression of LEA.92. In addition to the above model, another model system that consists of a non-tumorigenic immortalised MEC line (HuMl) and its transformed variant of the MuMl-TTu1 line, which is tumorigenic in nude mice, was also employed in this study (Ceyhan *et al.*, 1990).

### *Preparation of immunotolerogen and immunogen*

The established lines of human mammary epithelial cells (MCF.7 and MDA.MB.231) were cultured and washed with phosphate-buffered saline (PBS) as described previously (Imam *et al.*, 1985). The washed cells ($10^7$ cells $ml^{-1}$) were

Correspondence: S.A. Imam, Cancer Research Laboratory, USC School of Medicine, 1303 N. Mission Road, Los Angeles, CA 90033, USA.
Received 19 May 1993; and in revised form 14 September 1993.

lysed with 0.05 M Tris-HCl buffer, pH 7.5, containing 0.15 M sodium chloride, 1 mM phenylmethylsulphonyl fluoride, 0.5 mM chloromethyl-L-(2-phenyl-1-p-toluenesulphonamide) ethyl ketone and 0.5% (v/v) Nonidet P-40 (NP-40) (solubilisation buffer) on ice for 15 min. The lysates were centrifuged at 35,000 g and 4°C for 30 min. The supernatants containing NP-40-solubilised materials from the two cell lines were pooled (50%, v/v) and used for immune tolerisation, enzyme-linked immunosorbent assay (ELISA) or immunoprecipitation.

The normal breast tissues were obtained from two young women undergoing reduction mammoplasty with no evidence of abnormality. The tissue samples were pooled and minced, washed with cold PBS, suspended in solubilisation buffer without NP-40 (10 g, w/v) and homogenised on ice. The homogenate was centrifuged at 500 g and 4°C for 15 min. The supernatant was removed and was successively centrifuged at 35,000 g and 4°C for 30 min and at 100,000 g for 1 h. The pellet was recovered and solubilised in the solubilisation buffer, centrifuged at 100,000 g and 4°C for 1 h. The supernatant containing NP-40-solubilised materials was utilised for immunisation, ELISA and immunoprecipitation.

*Generation of monoclonal antibodies by a modified immunisation procedure*

Our previous approach to generate antibody against an antigen present on one cell type (A) but absent from another (B), against a background of numerous strong antigens common to both cell types (AB), has been the use of a procedure that consists of tolerisation of mice with cells of type B, followed by immunisation with cells of type A (Imam *et al.*, 1990*a*). Achievement of tolerance was evaluated by immunohistochemical methods, testing sera from mice against the tolerogen (MCF.7 and MBA.MD.231 combined). Tolerised mice, showing absence of serum antibodies against the tolerogen, were subsequently immunised with extracts of breast tissue containing normal breast cells (immunogen). Evidence of antibody production was sought by contrasting positive reactivity for normal MEC with absence of reactivity for their malignant counterparts by immunohistochemical methods. The spleen cells from a mouse showing evidence of serum antibody with these characteristics, and strong reactivity, was subsequently used for hybridisation and production of monoclonal antibodies (Imam *et al.*, 1990*a*,*b*). The initial screening of hybridoma supernatants was performed using freshly frozen tissue sections containing normal or malignant MEC using an immunohistological staining method described previously (Imam *et al.*, 1990*a*,*b*). Supernatants containing antibodies with no reactivity against many cell types in tissue sections were rejected. A small number of wells with hybrids secreted antibody that showed strong reactivity with the normal cells, but lacked reactivity against mammary carcinoma cells in tissue sections. These hybrids were repeatedly subcloned, until one clone, producing consistently high levels of monoclonal antibody with the above properties, was selected for detailed study. This antibody was termed anti-luminal epithelial antigen, LEA.92, to indicate its reactivity and an apparent molecular weight of the target antigen. Anti-LEA.92 antibody was purified and also labelled with biotin as described previously (Imam *et al.*, 1985). Double immunodiffusion studies with goat antibodies to a subclass of mouse immunoglobulin revealed that anti-LEA.92 antibody is an IgG1 immunoglobulin with a kappa light chain.

*Preparation and staining of tissue sections and cell lines*

An indirect unlabelled primary antibody method was used for the localisation of antigen with the specific antibody-utilising cell lines and tissue sections as described previously (Imam *et al.*, 1990*a*,*b*). The specificity and pattern of reactivity of anti-LEA.92 antibody were identical in frozen and formalin-fixed tissue sections, leading to a preference for the latter, based upon superior morphology and availability of a large number of tissue specimens. For each experiment, negative controls were performed to ensure the specificity of the reaction: these included the use of specific antibody following absorption with the immunogen, non-immune mouse serum or an irrelevant antibody of the same immunoglobulin class in lieu of the specific antibody. The visual estimates of intensities were scored as follows: –, absence; 1 +, weak; 2 +, moderate; and 3 +, intense.

*Comparison of LEA.92 with other known antigens of epithelial cells*

Competitive immunocytochemically steric interference assays were performed, using immunocytological techniques (Imam *et al.*, 1985, 1990*b*), in order to compare and contrast the epitope recognised by anti-LEA.92 antibody with those of epithelial membrane antigen (EMA) (Heyderman *et al.*, 1979), milk fat globule membrane glycoprotein (MFGM-gp70) (Imam *et al.*, 1981, 1984), MFGM-gp155 (Imam *et al.*, 1982, 1986), human milk fat globule 1 (HMFG-1) (Arklie *et al.*, 1981), HMFG-2 (Burchell *et al.*, 1984) and a member of the keratin family, pan-keratin (Schlegel *et al.*, 1980). The acetone-fixed cytopreparations of 184 or 184A1 and 184B5 HME cells were incubated first with the unlabelled test antibodies that included the above antibodies, followed by incubation with biotinylated antibody to LEA.92. The remainder of the staining procedure was as described previously (Imam *et al.*, 1990*a*,*b*). Any change in the intensity of staining with reference to control preparation was recorded.

*Metabolic labelling of cells and preparation of cell lysate*

Non-tumorigenic normal (184), non-tumorigenic immortalised (184A1, 184B5) or tumorigenic malignant (184A1N4-T-D10, MCF.7, MDA-MB-231, MDA-MB-468 or ZR.75) (MEC) lines were grown as monolayer cultures in 75 $mm^2$ tissue culture flasks and intrinsically labelled when cultures were still subconfluent. The cells were labelled for 24–48 h with 2 mCi of either [$^3$H]leucine or [$^3$H]galactosamine (110 Ci $mmol^{-1}$) per flask of leucine- or galactosamine-free DMEM respectively. Following incubation, the cells were washed three times and lysed with 0.05 M Tris-HCl buffer, pH 7.5, containing 0.15 M sodium chloride, 0.5% (v/v), 0.5% NP-40 (w/v) sodium deoxycholate, 1 mM phenylmethylsulphonyl fluoride and 0.5 mM chloromethyl-L-(2-phenyl-1-p-toluenesulphosamide) ethyl ketone on ice for 15 min. The lysates were centrifuged at 40,000 g and 4°C for 10 min. The supernatants containing detergent-solubilised materials were subsequently used for immunoprecipitation.

*Sodium dodecyl sulphate-polyacrylamide gel electrophoretic analysis of immunoprecipitants*

The radiolabelled cell lysates (approximately 400 ng of protein containing $5 \times 10^7$ c.p.m.) were mixed with 100 µl of either anti-LEA.92 antibody (0.1 mg $ml^{-1}$) or anti-LEA.92 antibody preabsorbed with the immunogen, non-immune mouse serum or an irrelevant monoclonal antibody of the same immunoglobulin class, as described previously (Imam *et al.*, 1990*a*,*b*). The latter antibody served as a negative control.

The materials immunoprecipitated with anti-LEA.92 antibody were subsequently analysed under chemically non-reducing (in the absence of 2-mercaptoethanol) or reducing conditions by sodium dodecyl sulphate-polyacrylamide gel electrophoresis (SDS-PAGE). The solubilised materials were subjected to electrophoresis in 7.5% polyacrylamide slab gels in the presence of SDS by the method of Laemmli (1970) and subjected to fluorography (Imam *et al.*, 1985).

### Results

### *Immunocytological localisation of LEA.92 in cell lines*

In order to test the *in vitro* specific expression of LEA.92, several human breast cell lines were used in an indirect immunocytochemical staining method. In addition to using the widely used lines of malignant mammary epithelial cells, a model system that consists of normal (184), the immortalised (184A1, 184B5 or HuMl) and the oncogenically transformed (184A1N1-T-D10, HuMl-TTu1) human mammary epithelial cell lines was analysed for the expression of LEA.92.

Non-tumorigenic normal (184) or immortalised (184A1, 184B5, HuMl) MEC lines showed a strong expression of LEA.92 of their plasma membrane. A representative reactivity with the 184A1 line is shown in Figure 1. The preabsorbed anti-LEA.92 antibody, non-immune mouse serum or an irrelevant monoclonal antibody of the same immunoglobulin class failed to react with the target cells. In contrast, the oncogenically transformed tumorigenic MEC lines (184A1N4-T-D10 or HuMl-TTu1) failed to exhibit a detectable expression of LEA.92 (Table I). In addition, established tumour cell lines derived from carcinomas of breast (Table I), cervix, colon, liver, lung, pancreas, kidney, stomach and thyroid or melanoma and haematopoietic cell lines (result not shown) also failed to exhibit a detectable amount of LEA.92 expression.

Specificity of expression of LEA.92 on the non-tumorigenic MEC lines was compared with other known epithelial antigens, which included human milk fat globule membrane antigens (e.g. epithelial membrane antigen, EMA; human milk fat globule membrane glycoproteins, MFGM-gp70; MFGM-gp155; human milk fat globule 1, HMFG-1; HMFG-2; epithelium-specific pan-keratin; and receptor for epidermal growth factor, EGF). The immunoblocking assays showed that the antigenic binding site for anti-LEA.92 antibody was not blocked by other antibodies, suggesting that the epitope recognised by the antibody is distinct. The antigen recognised by anti-LEA.92 is also different with respect to its molecular weight, as shown in Figure 2. Furthermore, in contrast to anti-LEA.92 antibody, antibodies to the above antigens reacted with all the cell lines included in Table I.

### *Localisation of antigen in tissue sections with anti-LEA.92 antibody*

In breast tissues from normal individuals, LEA.92 was expressed predominantly on the apical plasma membrane of luminal epithelial cells lining the ducts (Figure 3a). As in normal breast, benign breast diseases, such as fibroadenoma or hyperplasia, exhibited the expression of LEA.92. In contrast, invasive mammary carcinoma cells of both infiltrating ductal and infiltrating lobular types failed to exhibit a detectable amount of LEA.92 (Figure 3b and Table II). In extramammary tissue, a characteristically similar pattern of expression of LEA.92 was also observed in various normal glandular epithelial cells, which included cervix, colon, lung, pancreas and stomach. As in breast, the expression of LEA.92 was detectable on normal cells of the above organs, whereas corresponding malignant epithelial cells in tissue were consistently negative (Table II). Again, as in the cell line, antibodies to other known epithelial antigens failed to discriminate normal from malignant mammary or extramammary epithelial cells in the above tissues.

### *Characterisation of the antigen recognised by anti-LEA.92 antibody*

Antigen specifically recognised by anti-LEA.92 antibody was analysed by immunoprecipitation, SDS-PAGE and fluorography. Sources of [$^3$H]galactosamine- or [$^3$H]leucine-labelled antigen preparations included NP-40 extracts of the established (MCF.7 and MDA.MB.231), the oncogenically transformed (184A1N4-T-D10 or HuMl-TTu1), the immortalised (184A1, 184B5, HuMl) and the normal (184) mammary epithelial cell lines. Fluorigraphic analysis of the immunoprecipitate obtained by incubation of [$^3$H]galactosamine or [$^3$H]leucine-labelled lysates from the normal (184) or immortalised (184A1 or 184B5) MEC lines with anti-LEA.92 antibody on SDS–PAGE under reducing conditions showed a component with an apparent molecular weight of 92 kDa (Figure 2, lane B). The results clearly suggest that LEA.92 is glycosylated. The apparent molecular weight of the antigen from these different sources as recognised by the antibody was identical (results not shown). The antibody showed no detectable immunoprecipitable component from the lysate of MCF.7, MDA.MB.231, 184A1N4-T-D10 or HuMl-TTu1, complementing the results obtained in the immunocytological staining of these cell lines with the antibody as shown in Table I. Chemical reduction with 2-mercaptoethanol of immunoprecipitants had no effect on the migration of the antigen (results not shown). Furthermore, the preabsorbed anti-LEA.92 antibody, non-immune mouse

**Figure 1** Reactivity of anti-LEA.92 antibody to immortalised mammary epithelial cell line (184A1). The cytopreparations of the cell line (184A1) were fixed with cold acetone and incubated with anti-LEA.92 antibody. The antibody shows strong reactivity predominantly with the cell surface. Following the immunostaining, the cells were counterstained with Mayer's haematoxylin (original magnification × 200).

**Table I** Reactivity of anti-LEA.92 antibody with human mammary epithelial cells by an indirect immunocytological staining method[a]

| *Cell line* | *Reactivity with antibody to LEA.92* |
|---|---|
| *Non-tumorigenic in nude mice* | |
| 184 | + + |
| 184A1 | + + |
| 184B5 | + + |
| HuMI | + + |
| *Tumorigenic in nude mice* | |
| 184A1N4-T-D10 | – |
| HuMI-TTu1 | – |
| MCF.7 | – |
| ZR.75.1 | – |
| ZR.75.30 | – |
| MDA.MB.157 | – |
| MDA.MB.231 | – |
| MDA.MB.468 | – |
| HS 578 T | – |
| HS 746 T | – |
| HS 766 T | – |
| BT-20 | – |
| BT-483 | – |
| BT-549 | – |
| SK-BR-2III | – |
| SK-BR-3 | – |

[a]Samples were scored for intensity on a scale from – to + +: –, absence of staining; +, weak staining; + +, intense staining.

**Figure 2** Sodium dodecyl sulphate-polyacrylamide gel electrophoresis analysis of component immunoprecipitated by anti-LEA.92 antibody. Component immunoprecipitated by the antibody and [$^3$H]galactosamine-labelled lysate of the non-tumorigenic immortalised (184A1) mammary epithelial cell line, lane B. The radiolabelled lysate of 184A1 was also subjected to immunoprecipitation with the antibody preabsorbed with the immunogen as shown in lane C. Molecular weight standards are shown in lane A.

**Figure 3** Binding pattern of anti-LEA.92 antibody to mammary epithelial cells in formalin-fixed and paraffin-embedded tissue sections by an indirect immunohistological staining method. The sections were counterstained with Mayer's haematoxylin. The stromal components were consistently negative. **a**, Lactating duct. The antibody exhibited a homogeneous and predominant reactivity with apical plasma membrane of the lactating mammary epithelial cells (original magnification × 200). **b**, Normal (uninvolved) and infiltrating ductal carcinoma of breast. The uninvolved duct at the upper left side (short arrow) showed strong reactivity of predominantly apical plasma membrane with the antibody, whereas the surrounding malignant cells are completely unreactive (original magnification × 150).

**Table II** Immunohistological localisation of LEA.92 in formalin-fixed and paraffin-embedded normal or neoplastic mammary and extramammary tissue sections

| *Histology* | *No. of specimens studied* | *No. of specimens stained* | *Intensity of staining* |
|---|---|---|---|
| Breast, normal | 10 | 10 | 2 + to 3 + |
| Breast, benign | | | |
| Hyperplasia | 15 | 15 | 1 + to 3 + |
| Fibroadenoma | 15 | 12 | 1 + to 3 + |
| Breast, adenocarcinomas | | | |
| Infiltrating lobular invasive | 30 | 0 | – |
| Infiltrating ductal invasive | 50 | 0 | – |
| Medullary invasive | 10 | 0 | – |
| Mucoid invasive | 10 | 0 | – |
| Metastatic to regional lymph node | 10 | 0 | – |
| Cervix | | | |
| Normal | 4 | 4 | 2 + to 3 + |
| Adenocarcinoma | 4 | 0 | – |
| Colon | | | |
| Normal | 4 | 4 | 2 + to 3 + |
| Adenocarcinoma | 4 | 0 | – |
| Lung | | | |
| Normal | 4 | 4 | 1 + to 2 + |
| Adenocarcinoma | 4 | 4 | – |
| Pancreas | | | |
| Normal | 4 | 4 | 2 + tp 3 + |
| Adenocarcinoma | 2 | 0 | – |
| Stomach | | | |
| Normal | 4 | 4 | 2 + to 3 + |
| Adenocarcinoma | 4 | 0 | – |
| Cutaneous melanoma | 4 | 0 | – |
| Spleen, normal | 4 | 0 | – |
| Lymph node, normal | 4 | 0 | – |
| Non-Hodgkin's lymphomas | 4 | 0 | – |
| Hodgkin's disease | 4 | 0 | – |

Sections were scored for intensity on a scale from – to 3 +; –, absence of staining; 1 +, weak staining; 2 +, moderate staining; 3 +, intense staining.

serum or an irrelevant monoclonal antibody of the same immunoglobulin class was non-reactive with the previously positive lysates of [$^3$H]galactosamine- or [$^3$H]leucine-labelled MEC lines (Figure 2, lane C).

### Discussion

In order to reduce the probability of obtaining antibodies to common immunodominant antigens present on normal mammary epithelial cells and their malignant counterparts, a method of immune tolerisation/immunisation was used to generate monoclonal antibodies. The method in principle favours production, and an enhanced detection, of antibodies to antigens that are present in small amounts in cells, or are of intrinsically low immunogenicity (Imam *et al.*, 1990*a*). In the present study, this approach was applied in an attempt to develop antibodies with specificity for antigens present on normal cells and absent in the corresponding malignancy. Consequently, immune tolerance to antigens of human mammary epithelial carcinoma cells (MCF.7 and MDA.MD.231 combined) was induced in neonatal mice prior to subsequent immunisation with normal breast cells. Finally, a clone producing a monoclonal antibody with the above property was selected for further study. The antibody recognised an antigen on normal MEC lines and was termed luminal epithelial antigen with an apparent molecular weight of 92 kDa (LEA.92).

The cells in normal breast tissue as well as benign breast disease, such as fibroadenoma or hyperplasia, expressed LEA.92, whereas invasive mammary carcinoma cells of both infiltrating ductal and lobular types were negative. The results obtained with staining of tissue sections suggest a loss of expression of LEA.92 in invasive primary carcinoma cells of mammary or extramammary tissues. A detailed study using a large number of cases is warranted to map precisely the expression of LEA.92 during the progression of oncogenesis by incorporating tissue samples from patients with various types of dysplasia and *in situ* carcinomas of mammary and extramammary tissues.

In order to study the significance of down-regulation of LEA.92 in the oncogenesis of breast, an *in vitro* model that consists of various steps of malignant transformation of mammary epithelial cells has been adopted. During the preliminary study, the results using the model system indicate a correlation between the absence of LEA.92 expression and the development of tumorigenicity, complementing the results obtained with tissue.

The pattern of expression of LEA.92 on MEC in culture model systems mirrored those in tissues, as the glycoprotein was detected on the normal or immortalised MEC lines, which were non-tumorigenic in nude mice. In contrast, LEA.92 was undetectable on oncogenically transformed or established lines of mammary carcinoma cells, which were highly tumorigenic. There are probably several possibilities that can be attributed to the absence of reactivity of anti-LEA.92 antibody with the malignant cells in tissues or cell cultures. The most plausible among them, for the elimination of the epitope recognised by the antibody, can be attributed to a change in splicing or post-translational modifications of LEA.92. The possible variation in the sequence can be probed by the technique of polymerase chain reaction (PCR), once the cDNA sequence encoding LEA.92 is determined.

The effect of down-regulation of LEA.92 on the development of tumorigenicity *in vivo* of mammary epithelial cell lines is not yet known. Therefore, the biological role of LEA.92 in the process of transformation can conceivably be determined by incorporating experiments to determine whether inducing expression of the glycoprotein by transfection can suppress the tumorigenic phenotype of the malignant and non-expressing cells. Alternatively, it would seem at least equally valuable to transfect non-tumorigenic cells with antisense LEA.92 to determine whether suppression of the glycoprotein might cause tumorigenicity. These aspects are the subject of continuing study.

We wish to thank Dr Frank McCormick of Cetus Corporation, Emeryville, CA, for providing human mammary epithelial cell line, 184A1N4-T-D10. We also would like to express our thanks to Mr Nicholas Douglas for photography and Ms Sarah Olivo, Ms Esther Olivo and Ms Arianne Helenkamp for skilfully typing the manuscript.

This investigation was supported by a grant from the Jean Cross Memorial Fund.

### References

ALI, I.U., LIDEREAU, R., THEILLET, C. & CALLAHAN, R. (1987). Reduction to homozygosity of genes on chromosome 11 in human breast neoplasia. *Science*, **238**, 185–188.

ARKLIE, J., TAYLOR-PAPADIMITRIOU, J., BODMER, W., EGAN, M. & MILLIS, R. (1981). Differentiation antigens expressed by epithelial cells in the lactating breast are also detectable in breast cancers. *Int. J. Cancer*, **28**, 23–29.

BAKER, S.J., FEARON, E.R., NIGRO, J.M., HAMILTON, S.R., PREISINGER, A.C., JESSUP, J.M., VAN TUINEN, P., LEDBETTER, D.H., BARKER, D.F., NAKAMURA, Y., WHITE, R. & VOGELSTEIN, B. (1989). Chromosome 17 deletions and p53 gene mutations in colorectal carcinomas. *Science*, **244**, 217–221.

BURCHELL, J., WANG, D. & TAYLOR-PAPADIMITRIOU, J. (1984). Detection of the tumour-associated antigens recognized by the monoclonal antibodies HMFG-1 and two in serum from patients with breast cancer. *Int. J. Cancer*, **34**, 763-768.

CEYHAN, A., BRANDT, D., AAPRO, M. & GARCIA, I. (1990). *In vitro* differentiation of human mammary epithelial cells immortalised by microinjection of SV40 DNA. *Anticancer Res.*, **10**, 1397–1398.

CHEN, L.-C., DOLLBAUM, C. & SMITH, H.S. (1989). Loss of heterozygosity on chromosome 1q in human breast cancer. *Proc. Natl Acad. Sci.*, **86**, 7204–7207.

CLARK, R., STAMPFER, M.R., MILLEY, R., O'ROURKE, E., WALEN, K.H., KRIEGLER, M., KOPPLIN, J. & MCCORMICK, F. (1988). Transformation of human mammary epithelial cells by oncogenic retroviruses. *Cancer Res.*, **48**, 4689–4694.

ELIYAHU, D., MICHALOVITZ, D., ELIYAHU, S., PINHASI-KIMHI, O. & OREN, M. (1989). Wild-type p53 can inhibit oncogene-mediated focus formation. *Proc. Natl Acad. Sci.*, **86**, 8763–8766.

FRIEND, S.H., BERNARDS, R., ROGELJ, S., WEINBERG, R.A., RAPAPORT, J.M., ALBERT, D.M. & DRYJA, T.P. (1986). A human DNA segment with properties of the gene that predisposes to retinoblastoma and osteosarcoma. *Nature*, **323**, 643–646.

FRIEND, S.H., DRYJA, T.P. & WEINBERG, R.A. (1988). Oncogenes and tumor-suppressing genes. *N. Engl. J. Med.*, **318**, 618–622.

GENUARDI, M., TSIHIRA, H., ANDERSON, D.E. & SANDERS, G.F. (1989). Distal deletion of chromosome 1p in ductal cancer of the breast. *Am. J. Hum. Genet.*, **45**, 73–77.

HAMMOND, S.L., HAM, R.G. & STAMPFER, M.R. (1984). Serum-free growth of human mammary epithelial cells: rapid clonal growth in defined medium and extended serial passage with pituitary extract. *Proc. Natl Acad. Sci.*, **81**, 5435–5438.

HANSEN, M.F. & CAVENEE, W.K. (1987). Genetics of cancer predisposition. *Cancer Res.*, **47**, 5518–5527.

HARRIS, H. (1988). The analysis of malignancy by cell fusion: the position in 1988. *Cancer Res.*, **48**, 3302–3306.

HEYDERMAN, E., STEELE, K. & ORMEROD, M.G. (1979). A new antigen on the epithelial membrane: its immunoperoxidase localisation in normal and neoplastic tissue. *J. Clin. Pathol.*, **32**, 35–39.

IMAM, A., LAURENCE, D.J.R. & NEVILLE, A.M. (1981). Isolation and characterisation of a major glycoprotein from milk-fat-globule membranes of human breast milk. *Biochem. J.*, **193**, 47–54.

IMAM, A., LAURENCE, D.J.R. & NEVILLE, A.M. (1982). Isolation and characterisation of two individual glycoproteins from human milk-fat-globule membranes. *Biochem. J.*, **209**, 37–41.

IMAM, A., TAYLOR, C.R. & TOKES, Z.A. (1984). Immunohistochemical study of the expression of human milk-fat-globule membrane, glycoprotein 70. *Cancer Res.*, **44**, 2016–2020.

IMAM, A., DRUSHELLA, M.M., TAYLOR, C.R. & TOKES, Z.A. (1985). Generation and immunohistological characterisation of human monoclonal antibodies to breast carcinoma cells. *Cancer Res.*, **45**, 263–271.

IMAM, A., DRUSHELLA, M.M., TAYLOR, C.R. & TOKES, Z.A. (1986). Preferential expression of a Mr 155,000 milk-fat-globule membrane glycoprotein on lumenal epithelium of lobules in human breast. *Cancer Res.*, **46**, 6374–6379.

IMAM, A., STATHOPOULOS, E. & TAYLOR, C.R. (1990*a*). Generation and characterisation of a murine monoclonal antibody to cervical glandular epithelium using mice rendered tolerant to cervical squamous epithelium. *Hybridoma*, **9**, 157–166.

IMAM, A., STATHOPOULOS, E., HOLLAND, S.L. & TAYLOR, C.R. (1990*b*). Characterisation of a cell surface molecule expressed specifically on B and Hodgkin's cells. *Cancer Res.*, **50**, 1650–1658.

KLEIN, G. (1987). The approaching era of the tumor suppressor genes. *Science*, **238**, 1539–1544.

KNUDSON Jr, A.G. (1985). Hereditary cancer, oncogenes, and anti-oncogenes. *Cancer Res.*, **45**, 1437–1443.

LAEMMLI, U.K. (1970). Cleavage of structural protein during assembly of the head of bacteriophage T4. *Nature*, **227**, 680–686.

LEE, W.-H., BOOKSTEIN, R., HONG, F., YOUNG, L.-J., SHEW, J.-Y. & LEE, E.Y.-H.P. (1987). Human retinoblastoma susceptibility gene: cloning, identification and sequence. *Science*, **235**, 1394–1399.

LÜBBERT, M., MILLER, C.W., CRAWFORD, L. & KOEFFLER, H.P. (1988). p53 in chronic myelogenous leukemia. *J. Exp. Med.*, **167**, 873–886.

LUNDBERG, D., SKOOG, L., CAVENEE, W.K. & NORDENSKJÖLD, M. (1987). Loss of heterozygosity in human ductal breast tumors indicates a recessive mutation on chromosome 13. *Proc. Natl Acad. Sci.*, **84**, 2372–2375.

MACKAY, J., ELDER, P.A., STEEL, C.M. & FORREST, A.P.M. (1988). Allele loss on short arm of chromosome 17 in breast cancers. *Lancet*, **ii**, 1384–1385.

PATHAK, S. (1992). Cytogenetics of epithelial malignant lesions. *Cancer*, **70**, 1660–1670.

SCHLEGEL, R., BANKS-SCHLEGEL, S., MCLEOD, J.A. & PINKUS, G.S. (1980). Immunoperoxidase localisation of keratin in human neoplasms. *Am. J. Pathol.*, **101**, 41–48.

STAMPFER, M.R. (1985). Isolation and growth of human mammary epithelial cells. *J. Tissue Culture Methods*, **9**, 107–116.

STAMPFER, M.R. & BARTLEY, J.C. (1985). Induction of transformation and continuous cell lines from normal human mammary epithelial cells after exposure to benzo[*a*]pyrene. *Proc. Natl Acad. Sci.*, **82**, 2394–2398.

STAMPFER, M.R. & BARTLEY, J.C. (1988). In *Breast Cancer: Cellular and Molecular Biology*, Dickson, R. & Lippman, M. (eds). Martinus Nijhoff: Norwell, MA.

STANBRIDGE, E.J. (1985). A case for human tumor suppressor genes. *Bioessays*, **3**, 252–276.

STANBRIDGE, E.J. (1987). Genetic regulation of tumorigenic expression in somatic cell hybrids. *Adv. Virol. Oncol.*, **6**, 83–97.

STANBRIDGE, E.J. (1990). Human tumor suppressor genes. *Annu. Rev. Genet.*, **24**, 615–657.

TAKAHASHI, T., NAU, M.M., CHIBA, I., BIRRER, M.J., ROSENBERG, R.K., VINOCOUR, M., LEVITT, M., PASS, H., GAZDAR, A.F. & MINNA, J.D. (1989). p53: a frequent target for genetic abnormalities in lung cancer. *Science*, **246**, 491–494.

VOGELSTEIN, B., FEARON, E.R., KERN, S.E., HAMILTON, S.R., PREISINGER, A.C., NAKAMURA, Y. & WHITE, R. (1989). Allelotype of colorectal carcinomas. *Science*, **244**, 207–211.

WALEN, K.H. & STAMPFER, M.R. (1989). Chromosome analysis of human mammary epithelial cells at stages of chemical-induced transformation progression to immortality. *Cancer Genet. Cytogenet.*, **37**, 249–261.

WEINBERG, R.A. (1989). Oncogenes, anti-oncogenes, and the molecular base of multistep carcinogenesis. *Cancer Res.*, **49**, 3713–3721.

WEINBERG, R.A. (1991). Tumor suppressor genes. *Science*, **254**, 1138–1146.

YUEN-KAI, T.F., MURPHREE, A.L., T'ANG, A., QIAN, J., HINRICHS, S.H. & BENEDICT, W.F. (1987). Structural evidence for the authenticity of the human retinoblastoma gene. *Science*, **236**, 1657–1661.


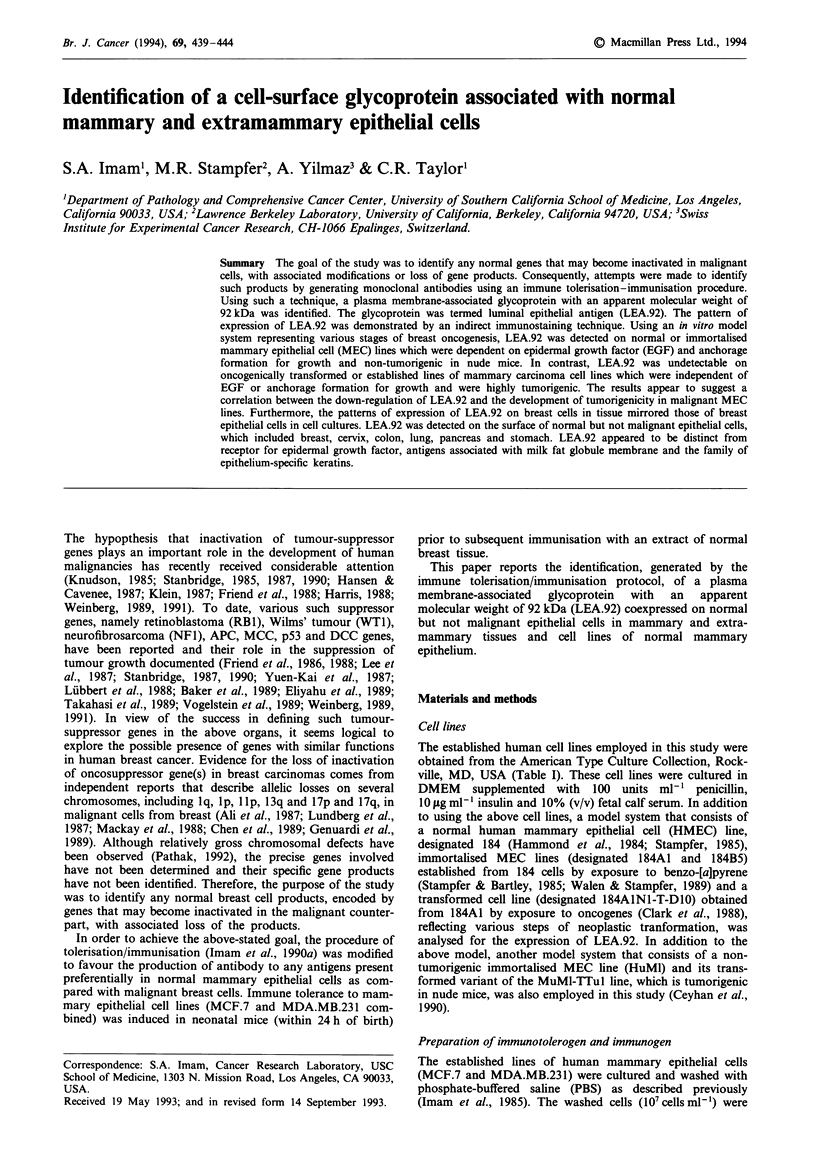

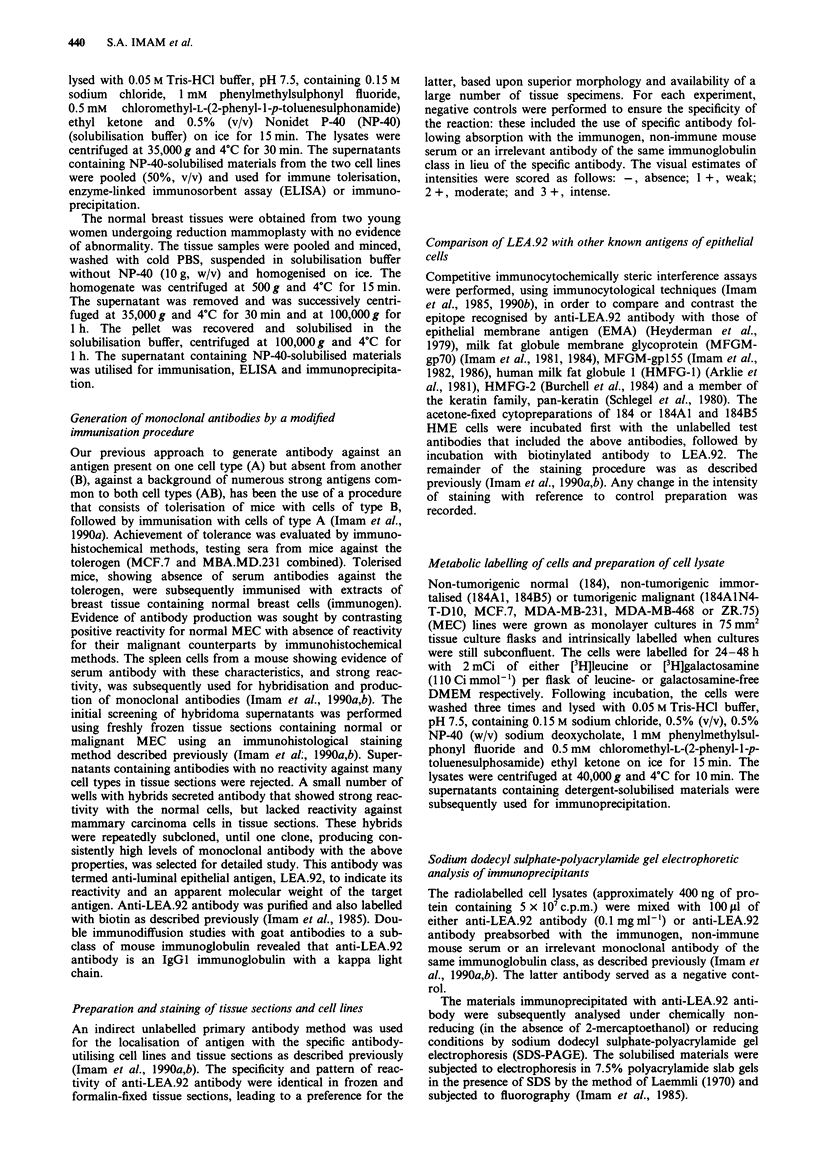

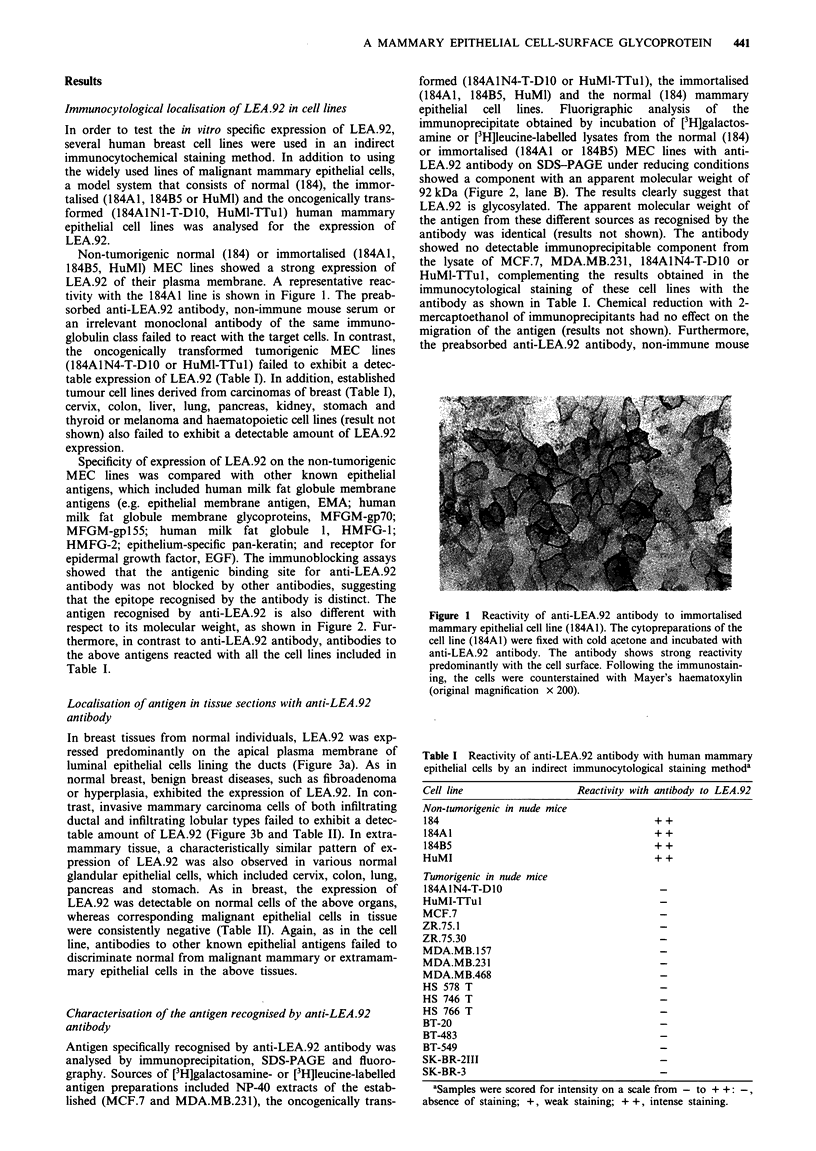

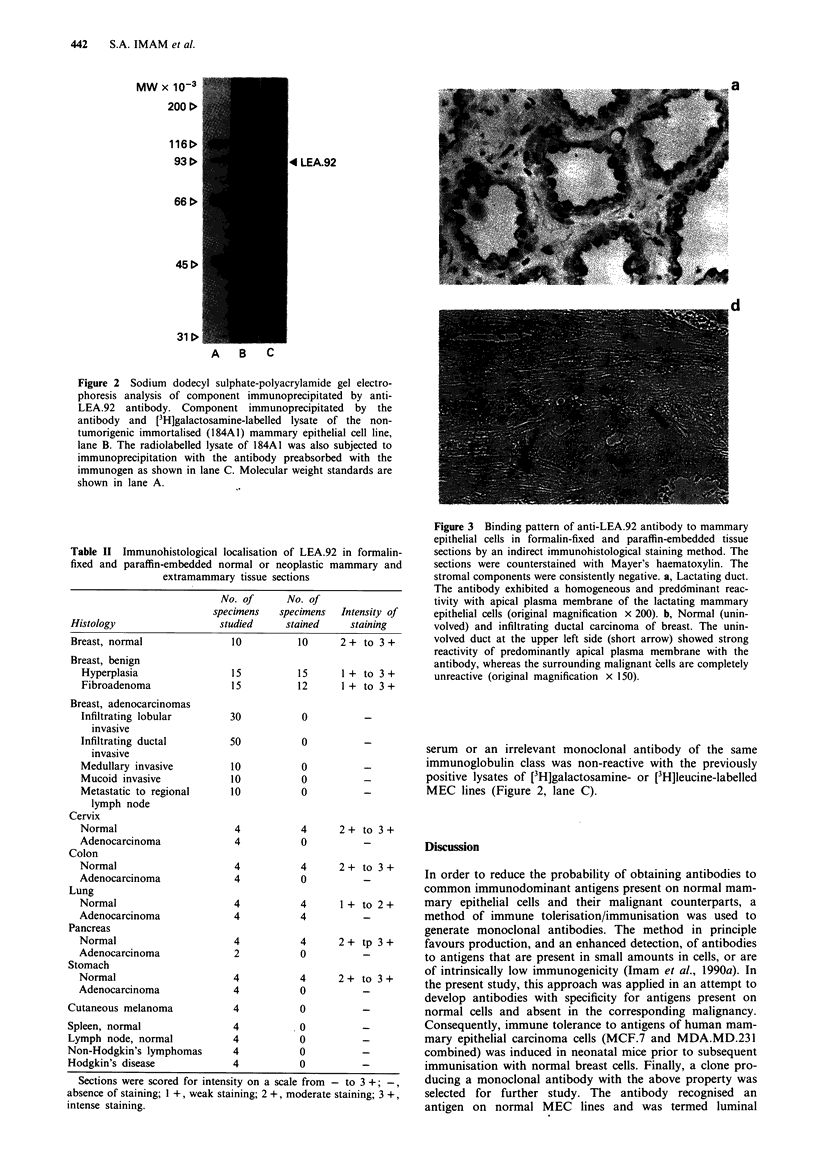

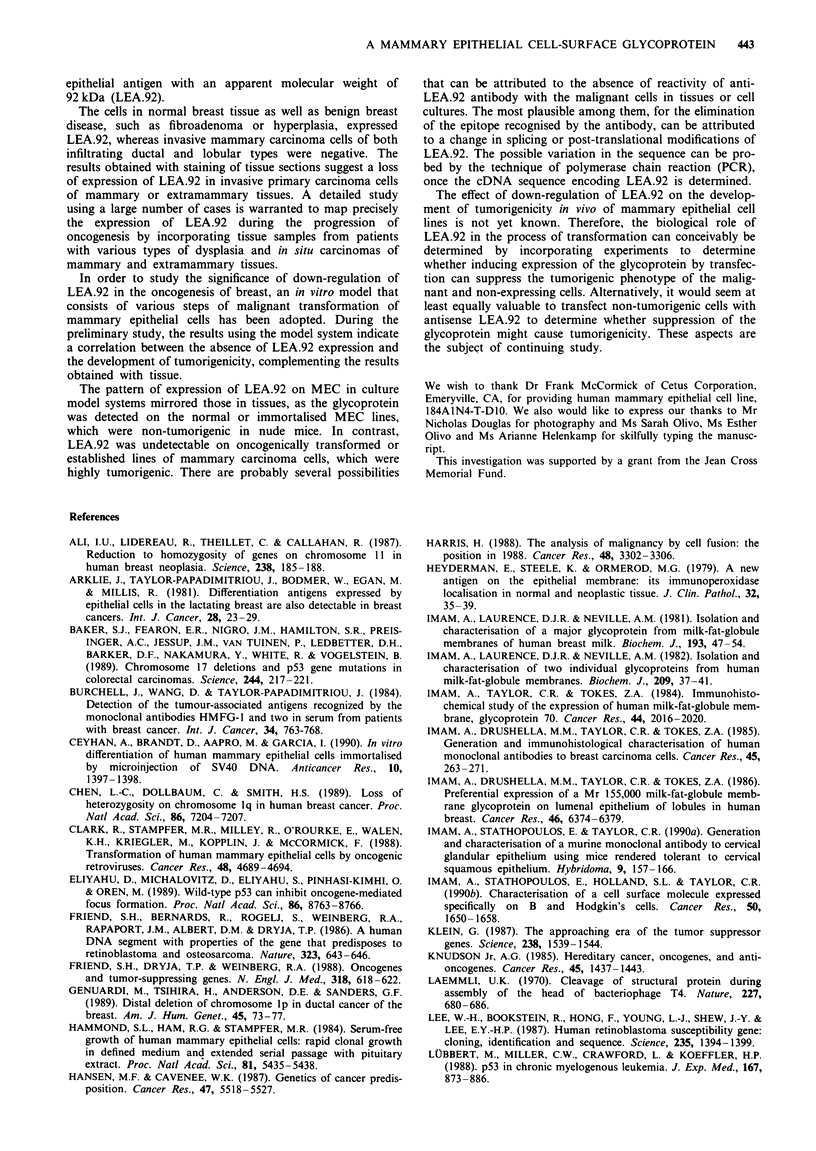

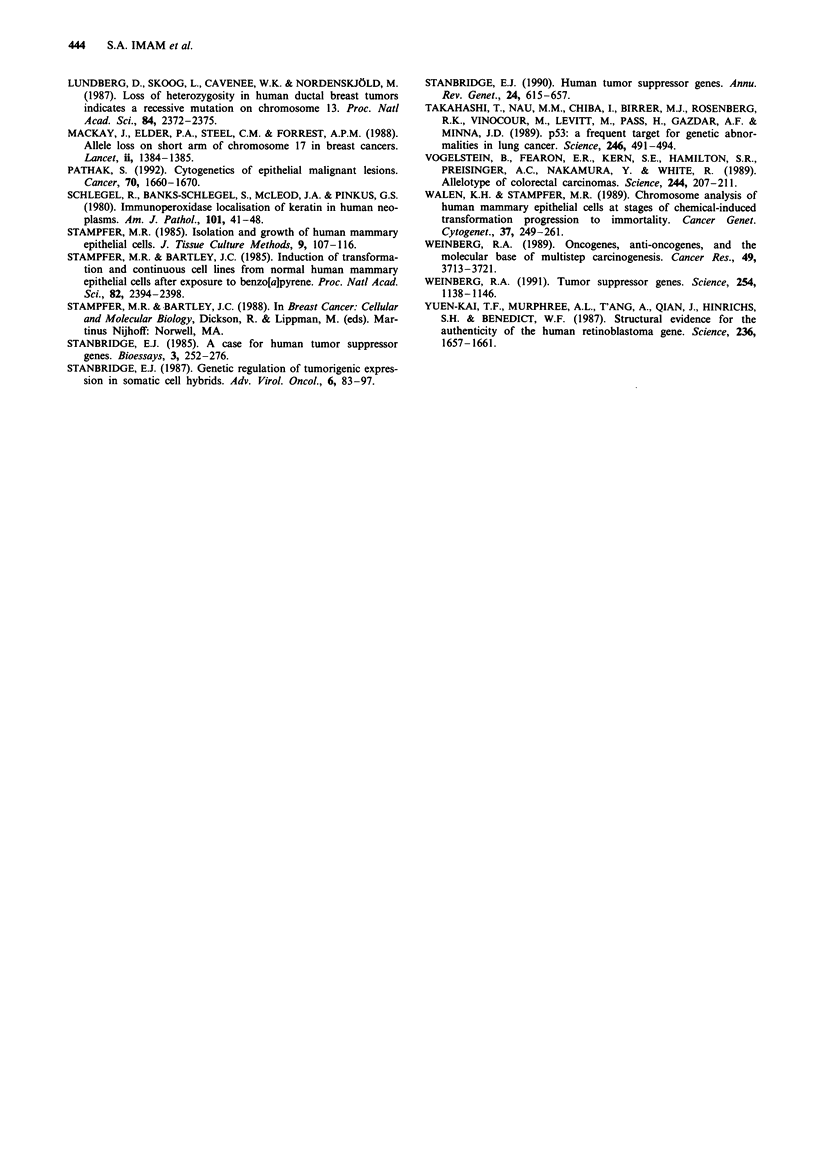


## References

[OCR_00614] Ali I. U., Lidereau R., Theillet C., Callahan R. (1987). Reduction to homozygosity of genes on chromosome 11 in human breast neoplasia.. Science.

[OCR_00619] Arklie J., Taylor-Papadimitrious J., Bodmer W., Egan M., Millis R. (1981). Differentiation antigens expressed by epithelial cells in the lactating breast are also detectable in breast cancers.. Int J Cancer.

[OCR_00627] Baker S. J., Fearon E. R., Nigro J. M., Hamilton S. R., Preisinger A. C., Jessup J. M., vanTuinen P., Ledbetter D. H., Barker D. F., Nakamura Y. (1989). Chromosome 17 deletions and p53 gene mutations in colorectal carcinomas.. Science.

[OCR_00632] Burchell J., Wang D., Taylor-Papadimitriou J. (1984). Detection of the tumour-associated antigens recognized by the monoclonal antibodies HMFG-1 and 2 in serum from patients with breast cancer.. Int J Cancer.

[OCR_00644] Chen L. C., Dollbaum C., Smith H. S. (1989). Loss of heterozygosity on chromosome 1q in human breast cancer.. Proc Natl Acad Sci U S A.

[OCR_00649] Clark R., Stampfer M. R., Milley R., O'Rourke E., Walen K. H., Kriegler M., Kopplin J., McCormick F. (1988). Transformation of human mammary epithelial cells by oncogenic retroviruses.. Cancer Res.

[OCR_00655] Eliyahu D., Michalovitz D., Eliyahu S., Pinhasi-Kimhi O., Oren M. (1989). Wild-type p53 can inhibit oncogene-mediated focus formation.. Proc Natl Acad Sci U S A.

[OCR_00660] Friend S. H., Bernards R., Rogelj S., Weinberg R. A., Rapaport J. M., Albert D. M., Dryja T. P. (1986). A human DNA segment with properties of the gene that predisposes to retinoblastoma and osteosarcoma.. Nature.

[OCR_00666] Friend S. H., Dryja T. P., Weinberg R. A. (1988). Oncogenes and tumor-suppressing genes.. N Engl J Med.

[OCR_00831] Fung Y. K., Murphree A. L., T'Ang A., Qian J., Hinrichs S. H., Benedict W. F. (1987). Structural evidence for the authenticity of the human retinoblastoma gene.. Science.

[OCR_00669] Genuardi M., Tsihira H., Anderson D. E., Saunders G. F. (1989). Distal deletion of chromosome Ip in ductal carcinoma of the breast.. Am J Hum Genet.

[OCR_00674] Hammond S. L., Ham R. G., Stampfer M. R. (1984). Serum-free growth of human mammary epithelial cells: rapid clonal growth in defined medium and extended serial passage with pituitary extract.. Proc Natl Acad Sci U S A.

[OCR_00680] Hansen M. F., Cavenee W. K. (1987). Genetics of cancer predisposition.. Cancer Res.

[OCR_00684] Harris H. (1988). The analysis of malignancy by cell fusion: the position in 1988.. Cancer Res.

[OCR_00688] Heyderman E., Steele K., Ormerod M. G. (1979). A new antigen on the epithelial membrane: its immunoperoxidase localisation in normal and neoplastic tissue.. J Clin Pathol.

[OCR_00709] Imam A., Drushella M. M., Taylor C. R., Tökés Z. A. (1985). Generation and immunohistological characterization of human monoclonal antibodies to mammary carcinoma cells.. Cancer Res.

[OCR_00715] Imam A., Drushella M. M., Taylor C. R., Tökés Z. A. (1986). Preferential expression of a Mr 155,000 milk-fat-globule membrane glycoprotein on luminal epithelium of lobules in human breast.. Cancer Res.

[OCR_00694] Imam A., Laurence D. J., Neville A. M. (1981). Isolation and characterization of a major glycoprotein from milk-fat-globule membrane of human breast milk.. Biochem J.

[OCR_00699] Imam A., Laurence D. J., Neville A. M. (1982). Isolation and characterization of two individual glycoprotein components from human milk-fat-globule membranes.. Biochem J.

[OCR_00727] Imam A., Stathopoulos E., Holland S. L., Epstein A. L., Taylor C. R. (1990). Characterization of a cell surface molecule expressed on B-lymphocytes and Hodgkin's cells.. Cancer Res.

[OCR_00721] Imam A., Stathopoulos E., Taylor C. R. (1990). Generation and characterization of a murine monoclonal antibody to cervical glandular epithelium using mice rendered tolerant to cervical squamous epithelium.. Hybridoma.

[OCR_00704] Imam A., Taylor C. R., Tökés Z. A. (1984). Immunohistochemical study of the expression of human milk fat globule membrane glycoprotein 70.. Cancer Res.

[OCR_00733] Klein G. (1987). The approaching era of the tumor suppressor genes.. Science.

[OCR_00737] Knudson A. G. (1985). Hereditary cancer, oncogenes, and antioncogenes.. Cancer Res.

[OCR_00741] Laemmli U. K. (1970). Cleavage of structural proteins during the assembly of the head of bacteriophage T4.. Nature.

[OCR_00746] Lee W. H., Bookstein R., Hong F., Young L. J., Shew J. Y., Lee E. Y. (1987). Human retinoblastoma susceptibility gene: cloning, identification, and sequence.. Science.

[OCR_00758] Lundberg C., Skoog L., Cavenee W. K., Nordenskjöld M. (1987). Loss of heterozygosity in human ductal breast tumors indicates a recessive mutation on chromosome 13.. Proc Natl Acad Sci U S A.

[OCR_00751] Lübbert M., Miller C. W., Crawford L., Koeffler H. P. (1988). p53 in chronic myelogenous leukemia. Study of mechanisms of differential expression.. J Exp Med.

[OCR_00764] Mackay J., Steel C. M., Elder P. A., Forrest A. P., Evans H. J. (1988). Allele loss on short arm of chromosome 17 in breast cancers.. Lancet.

[OCR_00769] Pathak S. (1992). Cytogenetics of epithelial malignant lesions.. Cancer.

[OCR_00773] Schlegel R., Banks-Schlegel S., McLeod J. A., Pinkus G. S. (1980). Immunoperoxidase localization of keratin in human neoplasms: a preliminary survey.. Am J Pathol.

[OCR_00782] Stampfer M. R., Bartley J. C. (1985). Induction of transformation and continuous cell lines from normal human mammary epithelial cells after exposure to benzo[a]pyrene.. Proc Natl Acad Sci U S A.

[OCR_00793] Stanbridge E. J. (1985). A case for human tumor-suppressor genes.. Bioessays.

[OCR_00801] Stanbridge E. J. (1990). Human tumor suppressor genes.. Annu Rev Genet.

[OCR_00805] Takahashi T., Nau M. M., Chiba I., Birrer M. J., Rosenberg R. K., Vinocour M., Levitt M., Pass H., Gazdar A. F., Minna J. D. (1989). p53: a frequent target for genetic abnormalities in lung cancer.. Science.

[OCR_00811] Vogelstein B., Fearon E. R., Kern S. E., Hamilton S. R., Preisinger A. C., Nakamura Y., White R. (1989). Allelotype of colorectal carcinomas.. Science.

[OCR_00816] Walen K. H., Stampfer M. R. (1989). Chromosome analyses of human mammary epithelial cells at stages of chemical-induced transformation progression to immortality.. Cancer Genet Cytogenet.

[OCR_00822] Weinberg R. A. (1989). Oncogenes, antioncogenes, and the molecular bases of multistep carcinogenesis.. Cancer Res.

[OCR_00827] Weinberg R. A. (1991). Tumor suppressor genes.. Science.

